# Novel Segmented Concentration Addition Method to Predict Mixture Hormesis of Chlortetracycline Hydrochloride and Oxytetracycline Hydrochloride to *Aliivibrio fischeri*

**DOI:** 10.3390/ijms21020481

**Published:** 2020-01-12

**Authors:** Huilin Ge, Min Zhou, Daizhu Lv, Mingyue Wang, Defang Xie, Xinfeng Yang, Cunzhu Dong, Shuhuai Li, Peng Lin

**Affiliations:** 1 Hainan Key Laboratory of Tropical Fruit and Vegetable Products Quality and Safety, Analysis and Testing Center, Chinese Academy of Tropical Agricultural Sciences, Haikou 571101, China; zhoumin05@yeah.net (M.Z.); hkwmy0815@163.com (M.W.); xdfang1@163.com (D.X.); yangxinf@sina.com (X.Y.); happylishuhuai@163.com (S.L.); 2 College of Plant Protection, Hainan University, Haikou 570228, China; czd@hainu.edu.cn; 3 Fujian SCUD Power Technology Co., Ltd., Fujian 350004, China; host-2008@163.com

**Keywords:** antibiotics, *Aliivibrio fischeri*, mixture hormesis, cross point hypothesis, concentration addition, isobole, co-toxicity coefficient, antagonism

## Abstract

Hormesis is a concentration-response phenomenon characterized by low-concentration stimulation and high-concentration inhibition, which typically has a nonmonotonic J-shaped concentration-response curve (J-CRC). The concentration addition (CA) model is the gold standard for studying mixture toxicity. However, the CA model had the predictive blind zone (PBZ) for mixture J-CRC. To solve the PBZ problem, we proposed a segmented concentration addition (SCA) method to predict mixture J-CRC, which was achieved through fitting the left and right segments of component J-CRC and performing CA prediction subsequently. We selected two model compounds including chlortetracycline hydrochloride (CTCC) and oxytetracycline hydrochloride (OTCC), both of which presented J-CRC to *Aliivibrio fischeri* (AVF). The seven binary mixtures (M1–M7) of CTCC and OTCC were designed according to their molar ratios of 12:1, 10:3, 8:5, 1:1, 5:8, 3:10, and 1:12 referring to the direct equipartition ray design. These seven mixtures all presented J-CRC to AVF. Based on the SCA method, we obtained mixture maximum stimulatory effect concentration (EC_m_) and maximum stimulatory effect (*E*_m_) predicted by SCA, both of which were not available for the CA model. The toxicity interactions of these mixtures were systematically evaluated by using a comprehensive approach, including the co-toxicity coefficient integrated with confidence interval method (CTCICI), CRC, and isobole analysis. The results showed that the interaction types were additive and antagonistic action, without synergistic action. In addition, we proposed the cross point (CP) hypothesis for toxic interactive mixtures presenting J-CRC, that there was generally a CP between mixture observed J-CRC and CA predicted J-CRC; the relative positions of observed and predicted CRCs on either side of the CP would exchange, but the toxic interaction type of mixtures remained unchanged. The CP hypothesis needs to be verified by more mixtures, especially those with synergism. In conclusion, the SCA method is expected to have important theoretical and practical significance for mixture hormesis.

## 1. Introduction

Hormesis is a concentration-response phenomenon characterized by low-concentration stimulation and high-concentration inhibition [1], which typically has a nonmonotonic CRC [2]. For example, herbicide 2,4-D in low concentration (10–30 μg/L) had significant stimulatory effect on plant root growth [3]. Antibiotics penicillin [4] and streptomycin [5] in low dose increased the mortality of mice infected with *Eberthella typhosa*, while reducing the mortality in higher dose. Calabrese et al. evaluated the response of *Escherichia coli* to antibacterial agents [6], the bacterial growth at concentrations below the toxic threshold was significantly greater than that in the controls, consistent with the characteristics of hormesis. A large number of examples had proved the existence of hormesis in nature [7,8,9].

Humans and other living organisms were always exposed to chemical mixtures in the environment [10]. We studied the combined effect of 10 ionic liquids (ILs) on luciferase, and observed that their mixtures presented a higher inhibitory effect, when all ILs components were in their maximum stimulatory effect concentration [11]. Therefore, the beneficial effects [12] in hormesis should be evaluated in the context of mixtures.

Antibiotics are substances obtained by culture of bacteria, fungi, actinomycetes and other microorganisms or by chemical synthesis for killing or inhibiting pathogenic microorganisms. Antibiotics played an important role in keeping humans and animals healthy. More and more evidence shows that antibiotics can induce hormesis on bacteria and other organisms [6]. Moreover, most of the environmental exposure concentrations of antibiotics were µg/L to ng/L or lower concentration [13], which was generally just in the concentration area of hormesis. At present, antibiotics and their mixtures can be found in water bodies, soil and other environmental systems [14], and the toxic interactions of antibiotics were also common [15]. Therefore, it is of great practical significance to study the mixture hormesis of antibiotics. Meanwhile, the evaluation of mixture hormesis is also more complicated and requires method innovation.

Antibiotics can induce hormesis to animals on the physiological level. Ciprofloxacin presented hormesis on the proliferation of rat astrocytes [16] and survival rate of human fibroblasts [17]. Antibiotics can induce hormesis on plant growth and algae reproduction. Chlortetracycline and oxytetracycline presented hormesis on the length of primary root, length of stalks and number of leaves of *Zea mays* [18]. The stimulatory effect of 10 µg/L tetracycline on duckweed can reach 26% [19]. Antibiotics also can induce hormesis on the physiological activities of microorganisms. Antibiotics in low concentration promoted yeast growing, while presenting inhibitory effects in high concentration, which were observed by the father of hormesis Schulz as early as 1888 [20]. Tetracycline showed a stimulatory effect on *Escherichia coli* in the range (0.015–0.030 µg/mL) far below the minimum inhibitory concentration (4 µg/mL), and the colony formation unit (CFU) increased to 141–121% relative to the blank [21]. Linares et al. [22] observed that tobramycin, tetracycline, and ciprofloxacin presented hormesis on the biofilm formation ability of *Pseudomonas aeruginosa*, suggesting that antibiotics were not only weapons against bacteria but also signaling molecules regulating the dynamic balance of microbial communities. Luminescent bacterium as an indicator organism was more and more used in environmental pollutant monitoring and ecotoxicology study. Deng et al. observed that sulfapyridine, sulfamethoxazole, sulfadiazine, sulfisoxazole, sulfamonomethoxine, and sulfachloropyridazine presented J-CRC to *photobacterium phosphoreum* [23]. Zou et al. observed that trimethoprim, sulfamethoxazole, sulphamethoxypyridazine and their mixtures presented J-CRC to *Vibrio fischeri* [24].

Concentration-response relationship is the central rule in toxicology, pharmacology, and environmental and ecological risk assessment [25]. The prediction of mixture toxicity can be attributed to calculating mixture CRC from single component CRC. Known CRC types reported in the literature generally included monotonic form of S-shaped, and nonmonotonic forms of J- or inverted J-shaped and U- or inverted U-shaped, etc. The hormesis was usually characterized by a nonmonotonic CRC. The inverted J-CRC and inverted U-CRC can be transformed into J-CRC and U-CRC through the conversion of activity and toxicity, and the U-CRC is part of the J-CRC, so all hormesis phenomenon can be characterized as J-CRC in essence. Therefore, prediction of mixture hormesis is the same thing as prediction of mixture J-CRC.

Two basic additive reference models of concentration addition (CA) and independent action (IA) were generally used to predict mixture toxicity [26]. Since the IA model would lose its probabilistic meaning when negative values (often referred to as a stimulatory response) were included [27], IA was once considered unfit to predict mixture J-CRC. At present, it is generally accepted that CA and IA merely as the working concept, which should not be added additional preconditions [28,29]. In particular, the application domain of IA should be used not only in S-CRC but also in J-CRC. Recently, IA had been successfully used in evaluating mixture hormesis of sulfonamide and quorum sensing inhibitor [30] and binary antibacterial chemicals [31] to *Aliivibrio fischeri*. However, CA is still the gold standard for additivity formulations [32]. Meanwhile, the CA model had the predictive blind zone (PBZ) for mixture J-CRC [11].

Then, under the J-CRC framework, the question is, how do you solve the PBZ problem? Belz et al. [33] extended the CA model in mathematical form by introducing the curvature parameter (λ) of the isobole to evaluate the mixture hormesis of parthenin and tetraneurin-A on *Lactuca sativa*. Ohisson et al. [34] calculated mixture *E*_m_ combining with CA prediction, and finally obtained mixture theoretical J-CRC through nonlinear fitting. Zou et al. [24] proposed the “six-point” approach to achieve the simulation of mixture whole J-CRC. Martin-betancor et al. [32] reported a prediction method for mixture inverted U-CRC. This method achieved good prediction for mixture U-CRC with the same effect sign (positive or negative), but the prediction for mixture J-CRC with both positive and negative effect cannot be completely satisfied. Qu et al. [35] developed an interpolation method based on the Delaunay triangulation and Voronoi tessellation to predict mixture hormesis.

However, most of the abovementioned methods to solve PBZ problem for CA were to introduce more parameters and assumptions, to use the fitting technique, or to use interpolation method, and the resulting model was too complex to be widely used and even difficult to understand. Therefore, it is urgent to establish a method with natural, direct, and simple application for predicting mixture whole J-CRC.

Can we convert nonmonotonic CRC into monotonic CRC? We planned to solve the PBZ problem according to the following steps. First, the component J-CRC was divided into the left and right segments on either side of the lowest point, which were fitted by monotonic function respectively. Then, CA can be used to predict the left and right segments of the mixture J-CRC respectively. Finally, predicting mixture whole J-CRC was achieved through the docking between left and right curves. This method can be called the segmented concentration addition (SCA) model.

## 2. Results and Discussion

### 2.1. Component J-CRC and Fitting

**Figure 1:**
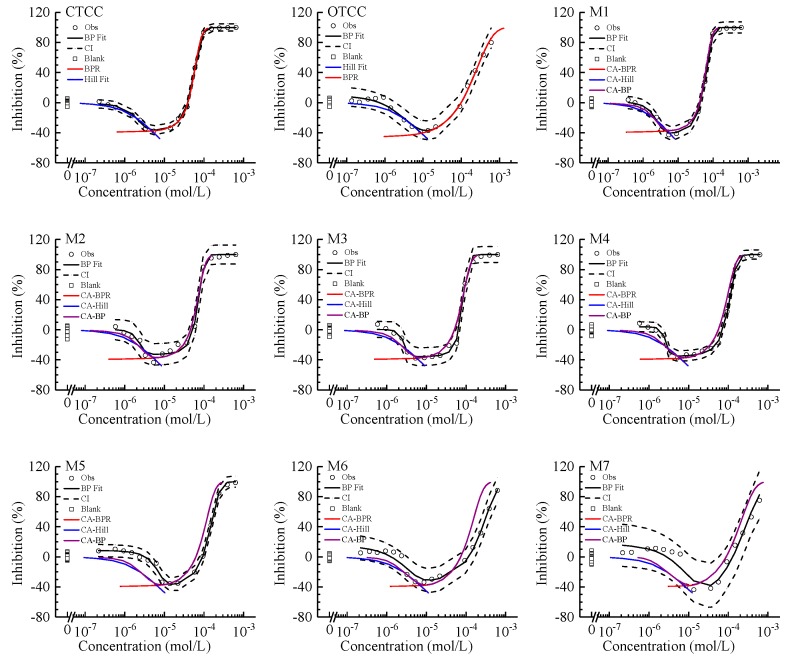
Concentration–response curves of chlortetracycline hydrochloride (CTCC), oxytetracycline hydrochloride (OTCC) and their mixtures inhibiting *Allivibrio fischeri*. Note: Square: blank control; Circle: observed data; Black dashed line: confidence interval; Black solid line: BP fit; Red line: BPR fit for single components or CA prediction based on BPR function for mixtures; Blue line: Hill fit for single components or CA prediction based on Hill function for mixtures; Violet line: CA prediction based on BP function; M1–M7 are the mixtures of CTCC and OTCC mixing with the molar ratios of 12:1, 10:3, 8:5, 1:1, 5:8, 3:10, and 1:12 respectively.

At the exposure time of 0.5 h, CTCC and OTCC inhibited AVF in a concentration-dependent manner with J-shaped CRC shown in Figure 1. The Biphasic (BP) regression model and the estimated parameters of J-CRC were summarized in Table 1. The J-CRC can be fitted by the five-parameter BP function with the root-mean-square error (RMSE) less than 0.05 and the coefficient of determination (*R*^2^) greater than 0.98. The variability of the blank control in the test was controlled within ±15%. There were two concentrations associated with the same stimulatory effect (−*x*%) in the two opposite phases of the J-CRC. We used EC_−_*_x_*_L_ and EC_−_*_x_*_R_ to denote the two concentrations on the left and right of the lowest point of the J-CRC. The maximum stimulatory effect (*E*_m_) was −36.9% for CTCC and −37.1% for OTCC. The representative indicators of effect concentration including EC_80_, EC_50_, EC_20_, EC_0_, EC_−20R_, EC_−30R_, EC_m_, EC_−30L_, EC_−20L_, and EC_−10L_ were shown in Table 1. The EC_50_ of OTCC was 4.6 times the EC_50_ of CTCC. This toxicity order also conformed the exposure dose order of chlortetracycline and oxytetracycline to *Zea mays* [18].

It is very important to fit the concentration-response data points. On the one hand, CRC, concentration-response functions (CRF), and the required representative effect concentrations can be obtained. On the other hand, for monotonic CRC, the mixture effect can also be predicted accurately based on the inverse function of CRF combining with the CA [26]. For S-CRC, two-parameter model such as Weibull and Logit [36] was generally accurate enough for description. For nonmonotonic CRC, excluding polynomial regression and support vector regression [37], some nonmonotonic functions including three-parameter Brain and Cousens model [38,39], four-parameter Schabenberger model [40,41] and Brain and Cousens model [42], and five-parameter Beckon model [43] were generally required. The most effective and typical model for describing J-CRC was the five-parameter functions, which were generally divided into two types including the addition form such as the Biphasic model [11] and Deng model [23] and multiplication form such as the Wang model [44,45] and Zhu model [46].

Biphasic model was good enough to describe J-CRC and just able to derive J-CRC left segment model (BPL) and J-CRC right segment model (BPR), so we used the biphasic function to fit the whole J-CRC. The mathematical expressions of BP, BPL, BPR and Hill were shown in the Section 3.2. Other five-parameter functions especially the Deng model [23] should also be able to derive the left and right segment models, but we did not verify this. In order to achieve the conversion from nonmonotonic CRC to monotonic CRC, we used the BP model to fit the whole J-CRC of CTCC and OTCC firstly. Then, we found that parameters *m*, *p* and *q* fitted by the BP function can be directly assigned to the BPR model, and the subsequently calculated curve according to BPR model was exactly consistent with the right segment of the BP curve as shown in Figure 1. This was a very important premise. After fitting the BP model, the BPR model can be directly obtained with no need to fit the right segment of J-CRC again.

**Figure 2:**
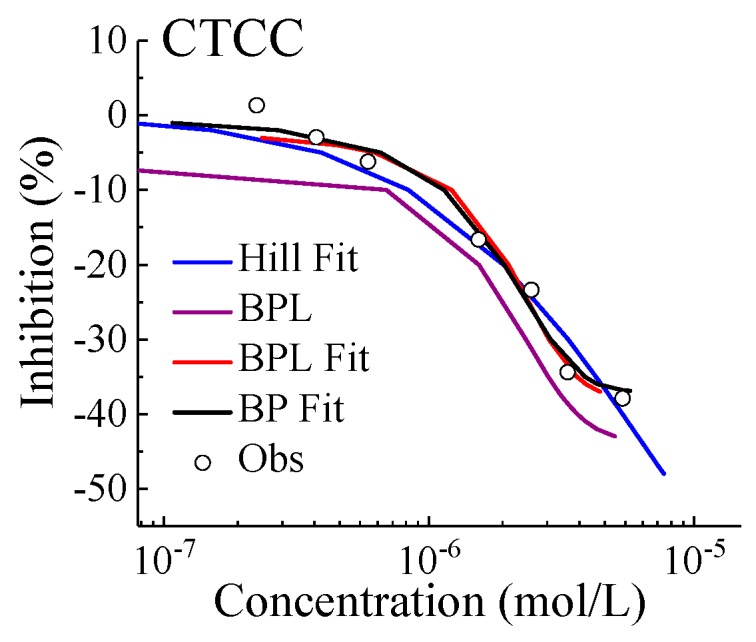
Modeling left segment of concentration–response curve of chlortetracycline hydrochloride (CTCC) inhibiting *Allivibrio fischeri*. Note: Circle: observed data; Black line: BP fit; Red line: BPL fit; Blue line: Hill fit; Violet line: BPL modeling based on BP parameters.

Unfortunately, parameters *m*, *a* and *b* fitted by the BP function cannot be used in the BPL model. It can be seen in Figure 2 for the CCTC left segment curve, when the fitted BP parameters were directly assigned to the BPL function, the subsequently calculated purple BPL curve deviated considerably from the black BP fit curve. Even more surprising was that the effect concentration calculated for OTCC by the same operation was negative, which we cannot explain yet. On the contrary, BPR can return the correct result for the right segment of J-CRC without depending on BRL. Moreover, the BP model can effectively and accurately describe the left segment of J-CRC, which should be the result of the joint action between BPR and BPL. According to the relational expression of $f_{\mathrm{BP}}=f_{\mathrm{BPL}}+f_{\mathrm{BPR}}-m$, we also tried other possible description forms for the left segment of J-CRC, such as E=−m/(1+10^(b × (C−a))), E=m−(1+m)/(1+10^(b × (C−a))), and E=m−(1−m)/(1+10^(b × (C−a))). The final modeling results showed that the effect concentration calculated from these equations was wrong and cannot be shown.

Then what happened if we fitted the left curve using BPL directly? The fitting results showed that the BPL model can effectively describe the left curve, but the bottom point was beyond the fitting range of BPL. More importantly, when BPL was combined with CA, it was usually unable to achieve the docking between the left and right curves, and the PBZ problem still cannot be solved. Fortunately, we finally found the famous Hill function to solve these problems. Hill function was not necessarily the best fit, but was the most efficient way to solve the PBZ problem for CA. The results of SCA prediction would be shown in the next section. The key point of fitting was that the observed lowest point should be included in BPR and Hill modeling simultaneously, which was conducive to achieving the docking between the left and right curves predicted by the CA.

### 2.2. Mixture J-CRC, CTC and Isobole

All seven binary mixtures (M1–M7) of CTCC and OTCC presented J-CRC shown in Figure 1, which were actually the reflection of the J-CRC of CTCC and OTCC. The J-CRC of CTCC, OTCC and their mixtures spanned at least three orders of magnitude on the concentration axis. The fitted CRC models and resulting parameters of these seven mixtures were given in Table 1. The observed J-CRC can be depicted by the BP function. In all cases, the *R*^2^s were greater than 0.91 and the RMSEs less than 0.12. Hill function was not used to fit the left segment of mixture J-CRC.

It can be seen from Figure 1 that, SCA and CA made basically identical predictions for the right segment of mixture J-CRC, which was understandable because BPR achieved a perfect representation for the right segment of the BP curve. When predicting the left segment of mixture J-CRC, there were some differences between SCA and CA, which was caused by the difference between Hill and BP functions when fitting the left segment of component J-CRC.

Under the SCA, the Hill prediction line and the BPR prediction line were effectively docking, and the predicted EC_m_ and *E*_m_ can be obtained subsequently. Relatively speaking, the CA model basically achieved the whole prediction of mixture J-CRC, although there was a very small PBZ notably in M7, which was caused by the *E*_m_ difference between CTCC and OTCC. Although they only had a 0.2% effect difference, which also led to the unpredictability of EC_m_ and *E*_m_ for mixtures. CA and SCA made a good prediction for J-CRC of M1–M4 mixtures, indicating that these four mixtures should basically act as additive action. However, for M5–M7 mixtures, the predicted J-CRCs by CA and SCA were significantly deviated from mixture observed J-CRC. In particular, the right segment of predicted CRC exceeded the CI of mixture observed CRC upwards for M5 and M6 mixtures, indicating a relatively obvious antagonistic action. Accurate toxicity interaction types would be determined using CTC and graphically illustrated with the isobole.

The CTC analysis results in Table 2 showed that CTCC and OTCC basically presented additive and antagonistic action, without synergistic action. Among the 63 judgment events in Table 2, antagonistic action appeared 26 times accounting for 41.3%, while additive action appeared 37 times accounting for 58.7%, indicating that the additivity played a slightly dominant role. On the other hand, the occurrence numbers of antagonism in 9 judgment events for each mixture were 9, 6, 4, and 0 for M5, M4, M3, and M1 respectively. The molar ratios of CTCC:OTCC in these four mixtures were 5:8, 1:1, 8:5, and 12:1 respectively, which proved well the equimolar ratio hypothesis proposed recently [29]. Although this hypothesis was initially developed based on binary enantiomer mixtures, it was suggested that this hypothesis may have a degree of universality for binary antagonistic mixtures, and several examples fitting the hypothesis were also observed in reference [33].

**Figure 3:**
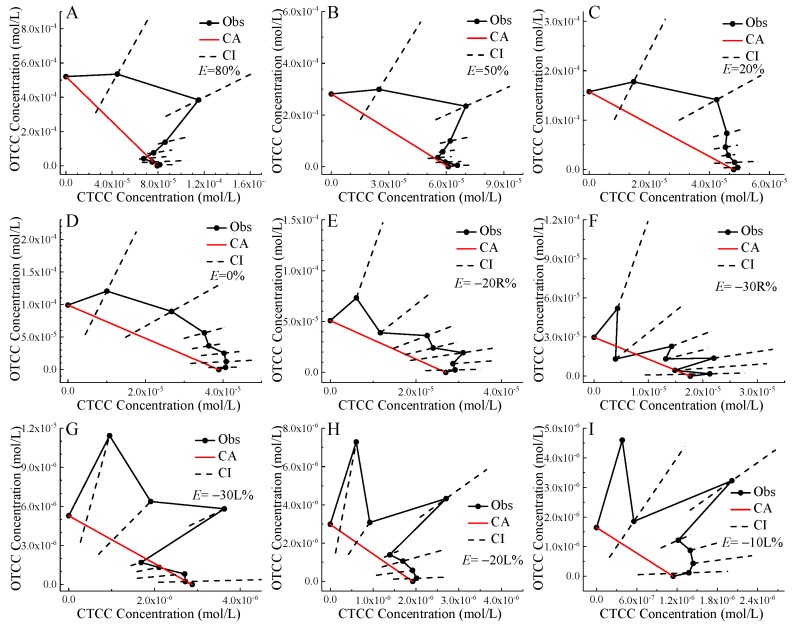
Isoboles of chlortetracycline hydrochloride (CTCC) and oxytetracycline hydrochloride (OTCC) to *Allivibrio fischeri* at 80% (**A**), 50% (**B**), 20% (**C**), 0% (**D**), −20R% (**E**), −30R% (**F**), −30L% (**G**), −20L% (**H**), and −10L% (**I**) effect levels. Note: Black point: observed equivalent point; Black solid line: observed isobole; Black dashed line: confidence interval; Red line: CA isobole; L and R refer to the left and right of the lowest point of the J-CRC respectively; Except for the two boundary points on the black solid line, the remaining seven points in line order from left to right correspond to CTCC:OTCC molar ratios of 12:1, 10:3, 8:5, 1:1, 5:8, 3:10, and 1:12 respectively.

Isobole was an effective tool for evaluating toxic interactions of binary mixtures within the S-CRC framework, such as the antagonism of chiral ILs enantiomer to *Aliivibrio fischeri* [29], synergistic antibacterial effect of ribavirin and disulfiram [47], and the estrogenic activity of UV filter mixtures to fish [48]. Only a few examples using isobole to evaluate mixture hormesis such as parthenin and tetraneurin-A on *Lactuca sativa* [33], it was worth noting that this study contained a non-equivalent isobole i.e., EC_m_ isobole. Nevertheless, most studies focused on the isobole for inhibitory or harmful effect, while using isobole for stimulatory effect had been rarely reported. Because CA was the intercept formula of isobole, CTC was deformation form of CA, and the CIs of equivalent point in isobole corresponded exactly to the CIs of CTC, so in principle CTC and isobole were completely equivalent. For example, the CTC values in blue very close to 100 in Table 2 corresponded the points right located on the isobole in Figure 3. The two methods would make the same judgment conclusion for the toxic interaction of binary mixtures. It can also be seen from the computational process based on the common basic elements including EC*_x_*_,mix_, *P_i_* and EC*_x_*_,*i*_. The advantage of isobole was very obvious, which was easy to use, intuitive and convenient [29]. The disadvantage of isobole was also very obvious, which cannot be used for the mixtures with more than three components, even if the isobole can be extended to the equivalent-surface for three-component mixtures [49]. The advantages of CTC were simple, intuitive, and quantitative [49]. In this sense, it was entirely possible for CTCICI to replace isobole, since they were equivalent and express the same meaning after all. However, for J-CRC, it was possible to face the situation that some effect concentrations had no CI. In this case, the CTCICI adaptable judgment rule for toxicity interaction types was proposed in the Section 3.3.

In the S-CRC framework, if CI was ignored, it was generally simple to determine the toxic interaction types based on mixture observed CRC and CA predicted CRC. When CA predicted CRC was coincided, above or below the mixture observed CRC, the mixture was additive, antagonistic or synergistic, respectively. But for J-CRC, the judgment based on S-CRC experience may result in errors such as the M5 mixture in Table 2 and Figure 1. There was a cross point (CP) between M5 mixture observed J-CRC and CA predicted J-CRC. On the right of the CP, the CA CRC above the observed CRC can be judged as antagonism according to the common sense. On the left of the CP, the CA CRC below the observed CRC can be judged as synergism according to the common sense, which was actually wrong however. Since both the two cases were actually antagonism according to CTCICI or isobole analysis.

This led to an interesting and important hypothesis of cross point. In the J-CRC framework, for antagonistic mixtures, there was generally a CP between mixture observed J-CRC and CA predicted J-CRC, the CA CRC was above mixture observed CRC on the right of the CP, while the CA CRC was below the mixture observed CRC on the left of the CP. For the CP, the corresponding concentration can be called the CP effect concentration (EC_CP_), and the corresponding effect can be called the CP effect (*E*_CP_). We also gave the two indexes of EC_CP_ and *E*_CP_ for M1–M7 mixtures in Table 1. For synergistic mixture, the CP hypothesis was in the contrary form, that the CA CRC was below mixture observed CRC on the right of the CP, while the CA CRC was above mixture observed CRC on the left of the CP. For both antagonistic and synergistic mixtures presenting J-CRC, the relative positions of observed and predicted CRCs on either side of the CP would exchange, but the toxic interaction type of mixtures remained unchanged. We were unable to provide an example of synergism for J-CRC yet now. Fortunately, references [30,31] provided the examples of synergistic mixtures presenting J-CRC, although the IA was selected as the additive reference model. However, the CP hypothesis for antagonistic and synergistic mixtures presenting J-CRC needs to be validated with more examples. It is important to note here that the CP hypothesis will not be limited to binary mixtures, but may also be applicable to mixtures with more components.

In the S-CRC framework, there may also be a CP between mixture observed CRC and CA predicted CRC, but the meaning of CP will be different, and the two sides of the CP will have different toxicity interaction types. In the J-CRC framework, the existence of the CP, the position transformation of mixture observed and CA predicted CRC, and mixture toxic interaction type remaining unchanged on either side of the CP may reflect some internal mechanism of hormesis.

Calabrese and Baldwin proposed that chemical hormesis can be produced in two ways including direct stimulation hormesis (DSH) and overcompensation stimulation hormesis (OCSH) [1,7]. When the homeostasis of the organism was disturbed by the toxic substances in low dose, the OCSH was an adaptive effect produced by the organism after a period of exposure.

### 2.3. Relationship between Mixture Toxicity and Component Molarity Proportions

**Figure 4:**
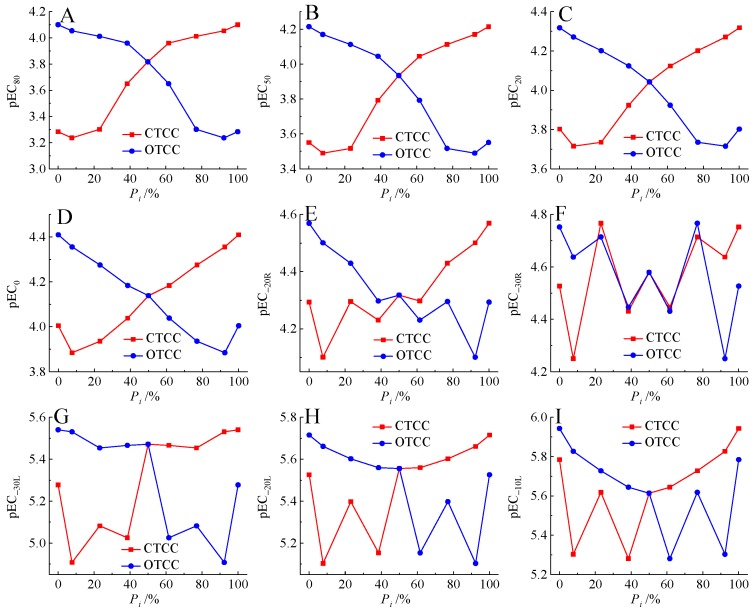
Relationship between mixture toxicities of pEC_80_ (**A**), pEC_50_ (**B**), pEC_20_ (**C**), pEC_0_ (**D**), pEC_−20R_ (**E**), pEC_−30R_ (**F**), pEC_−30L_ (**G**), pEC_−20L_ (**H**), pEC_−10L_ (**I**) and component molarity proportion (*P_i_*) of chlortetracycline hydrochloride (CTCC) and oxytetracycline hydrochloride (OTCC).

Previous studies indicated that there was biphasic U or inverted-U relationship between the binary mixture toxicity and the concentration proportion of components [29,49]. We studied the relationship between component molarity proportions and the toxicity of mixtures presenting J-CRC shown in Figure 4, the left and right boundary points corresponded to the single component (CCTC or OTCC) toxicity. Our results showed that there were 5 pairs of relatively obvious linear relationship between component molarity proportion (*P_i_*) and the mixture toxicities (pEC_80_, pEC_50_, pEC_20_, pEC_0_, pEC_−20R_) shown in Figure 4A–E. For these mixtures in each pair relationship, the more toxic component (CTCC) presented monotonically increasing relationship, the less toxic component (OTCC) presented monotonically decreasing relationship. This was understandable, because CTCC was smaller than OTCC for all nine effect concentrations including EC_80_, EC_50_, EC_20_, EC_0_, EC_−20R_, EC_−30R_, EC_−30L_, EC_−20L_, and EC_−10L_. However, this type of linear relationship was not observed in the remaining 4 toxicity indicators including pEC_−30R_, pEC_−30L_, pEC_−20L_, and pEC_−10L_ shown in Figure 4F–I. Among them, no obvious correlation relationship was observed between mixture toxicities (pEC_−30R_) and the *P_i_* of components shown in Figure 4F. Nevertheless, there was a significant jump for the mixture toxicity (pEC_−30L_, pEC_−20L_, pEC_−10L_) near the equimolar ratio of CCTC and OTCC shown in Figure 4G–I. This may in part be a reflection of the equimolar ratio hypothesis [29]. Interestingly, for the relationship between mixture toxicity and component concentration proportions, the nonmonotonic relationship was not observed in nonmonotonic J-CRC framework in the present study, while the nonmonotonic relationship was observed in monotonic S-CRC framework [29,49]. Whether this represents coincidence or a link is not clear but deserves further observations and investigation.

### 2.4. Significance, Limitation and Implications

The aim of this study was to establish an SCA method to predict mixture hormesis or mixture J-CRC and to solve the PBZ problem for CA in J-CRC framework. The SCA method retained the original essence of the CA model with simple and natural form and strong operability. The key to the successful prediction of SCA was to use the Hill function to fit the left segment of the J-CRC. The SCA method is an open platform and technology. In essence, the idea of segmented fitting for nonmonotonic CRC was proposed. The method itself had no termination or limitation. Other researchers can also use other functions to fit the nonmonotonic CRC piecewisely. The SCA method is expected to have a wide application prospect. The SCA can be used to evaluate mixture hormesis and predict the combined effect of estrogen, and endocrine disruptors with nonmonotonic CRC in the future. Moreover, the SCA method may have important enlightening significance for the toxicological and pharmacological studies, and the ecological and environmental risk assessment.

In the present study, the *E*_m_ values of CTCC and OTCC were very close, which led to the fact that the superiority of SCA over CA was not fully demonstrated. In the next study, it is best to choose synergistic components with a large *E*_m_ difference for further verification of SCA. Nevertheless, evaluating the toxicity interaction in mixture hormesis is challenging and only just now unfolding. The SCA predicted curve was not continuously differentiable at the docking point. This is a problem that needs further study and solution in the future.

In the present study, the stimulatory effect concentration on the left segment was generally lower than 1 × 10^−5^ mol/L (approximately 5 mg/L) for CTCC, OTCC and their mixtures. The concentration of antibiotics in the environment was generally lower than that concentration of 5 mg/L [13]. Antibiotics in environmental concentrations should all have the stimulatory effect. Then is there a certain relationship between hazardous events such as vibriosis in prawn culture, the outbreak of algal blooms in the lake and chemical hormesis from environmental pollution, which is worth further study.

## 3. Materials and Methods

### 3.1. Chemicals

Chlortetracycline hydrochloride (CTCC) and oxytetracycline hydrochloride (OTCC) were purchased from Ehrenstorfer GmbH (Augsburg City, Bavaria State, Germany). The chemical structures and related information of the two antibiotics were shown in Table 3. The stock solutions of CTCC and OTCC were prepared through dissolving them in the deionized water and stored in 4 °C refrigerator. The stock solutions of antibiotic mixtures were prepared through mixing the stock solutions of CTCC and OTCC according to their concentration ratios assigned.

### 3.2. Photobacterium Toxicity Test

The photobacterium *Aliivibrio fischeri* (Strain number 1H00019) was purchased from Marine Culture Collection of China (Xiamen City, Fujian province, China). The culture medium consisted of 1 g KH_2_PO_4_, 4.7 g Na_2_HPO_4_·12H2O, 0.3 g MgSO_4_·7H_2_O, 0.5 g (NH_4_)_2_HPO_4_, 30 g NaCl, 5.0 g yeast extract, 5.0 g tryptone, 3.0 g glycerin, and 1000 mL water, and was adjusted to pH 6.7 ± 0.3. The AVF was grown in the culture medium at 22 ± 1 °C by shaking (120 r/min) for 10–12 h for toxicity test.

The toxicities of single antibiotics and their mixtures were expressed as an inhibition of the AVF luminescence. According to the microplate toxicity analysis method [50], CTCC, OTCC, and their mixtures with 16 concentration series in at least four repeats and 24 controls were arranged on a microplate. Then, 100 μL AVF suspension was added into each well to reach the final volume of 200 μL. The relative light units (RLUs) of the AVF system exposed to single antibiotics and their mixtures were determined on Synergy 2 Multi-Mode Microplate Readers (BioTek Instruments, Winooski, VT, USA) with a 96-well white flat bottom microplate (Corning 3917) after 30 min of exposure at (26 ± 1) °C.

The effect (*E* of *x*%) of individual antibiotics and their mixtures was calculated according to Equation (1). The J-CRC was fitted by Biphasic (BP) function shown in Equation (2) using the least squares method [39]. The goodness of fit of statistical models was evaluated by *R*^2^ (coefficient of determination) and RMSE (root-mean-square error). As a quantitative measure of the uncertainty, the observation-based 95% CI was determined [51]. The BP model can derive two models of J-CRC left segment model (BPL) shown in Equation (3) and J-CRC right segment model (BPR) shown in Equation (5). Equations (4) and (6) were the inverse functions for Equations (3) and (5), respectively, and can be used to calculate the required effect concentrations. Equations (7) and (8) were the Hill function and its inverse function, and were used to fit the J-CRC left segment.

(1) $$E=1-\left(L/L_{0}\right)$$

(2) E=m−m/(1+10^(b × (C−a)))+(1−m)/(1+10^(q × (p−C)))

(3) E=m−m/(1+10^(b × (C−a)))

(4) C=a+1/b × log10(E/(m−E))

(5) E=m+(1−m)/(1+10^(q × (p−C)))

(6) C=p−1/q × log10((1−E)/(E−m))

(7) E=d × C/(k+C)

(8) C=k × E/(d−E)

Where *L*_0_ is the average of RLUs of controls, *L* is the average of RLUs of treatments, *E* is inhibitive effect of AVF luminescence, *C* is chemical concentration, *a* and *b* are the median and slope parameters in the left low concentration region, *p* and *q* are the median and slope parameters in the right high concentration region, *m* is the bottom parameter, *k* is the median parameter, and *d* is the top parameter.

### 3.3. Experimental Design and Toxicity Evaluation of Mixtures

The seven binary mixtures of CTCC and OTCC were designed according to the molar ratios of 12:1, 10:3, 8:5, 1:1, 5:8, 3:10, and 1:12 referring to the direct equipartition ray (EquRay) design [52]. We chose the molar ratio instead of the toxic unit ratio to verify the equimolar ratio hypothesis [29] and to increase the generality of the experimental design.

The model of CA shown in Equation (9) were used to predict the mixture effect concentration (EC*_x_*_,mix_) corresponding to the mixture x% effect, and the mixture predicted CRC was also presented [26]. The observed EC*_x_*_,mix_ was multiplied by the component concentration proportion (*P_i_*) to obtain the two partial concentrations which formed a point in the two-dimensional Cartesian coordinates. These points were connected to form the mixture observed isobole. When the CIs of mixture observed isobole were containing, above, or below mixture predicted isobole, the mixture was judged to present additive, antagonistic, or synergistic action, respectively. Judging toxicity interactions based on J-CRC and CA was more complicated, which would be discussed in detail in the Section 2.2.

On the other hand, the toxicity interactions of mixtures can be evaluated based on the CTCICI method developed recently [49] using components EC*_x_*_,*i*_, mixture observed EC*_x_*_,mix_ and its 95% CI. The CTC was computed according to Equation (10). When 100 was included in the CI of mixture CTC, the mixture presented additive action. When the CIs of mixture CTC were greater or smaller than 100, the mixture presented synergistic or antagonistic action, respectively. When one end of the CIs was missing, the other end of the CTC CI and the CTC formed a new form of CTC CI, which was also suitable for CTCICI discrimination rules. When both ends of the CTC CI were missing or 100 was not included in CTC CI with one end, CTCICI was reduced to CTC of the original form. The original discrimination rules of CTC were applicable, namely 80 ≤ CTC ≤ 120, CTC < 80, or CTC > 120 indicated additive, antagonistic, or synergistic action, respectively [53,54].

(9) $${\mathrm{EC}}_{x,\mathrm{mix}}=1/\sum_{i=1}^{n}\left(P_{i}/{\mathrm{EC}}_{x,i}\right)$$

(10) $$\mathrm{CTC}=100/\left({\mathrm{EC}}_{x,\mathrm{mix}}\times \sum_{i=1}^{n}\left(P_{i}/{\mathrm{EC}}_{x,i}\right)\right)$$

where *n* is the number of mixture components, EC*_x_*_,*i*_ is the concentration of *i*th component eliciting *x*% effect, EC*_x_*_,mix_ is the concentration of a mixture eliciting *x*% effect, *P_i_* is the concentration proportion of *i*th component in a mixture.

## 4. Conclusions

The mixture hormesis of chlortetracycline hydrochloride and oxytetracycline hydrochloride to *Aliivibrio fischeri* were explored. We proposed a segmented concentration addition (SCA) method to predict mixture whole J-shaped concentration-response curve (CRC) and to solve the problem of predictive blind zone (PBZ) for concentration addition (CA) model. We observed the cross point (CP) between observed J-CRC and CA predicted J-CRC for antagonistic mixture, and concluded that the relative positions of observed and predicted CRCs on either side of the CP would exchange, but the toxic interaction type of mixtures remained unchanged. Under the J-CRC framework, we reconfirmed the equimolar ratio hypothesis proposed recently, namely that mixture antagonism occurred most frequently at the equimolar ratio and its adjacent ratio, and there was a significant jump in mixture toxicity near the equimolar ratio.

## Figures and Tables

**Table 1:** Concentration–response model of chlortetracycline hydrochloride (CTCC), oxytetracycline hydrochloride (OTCC) and their mixtures inhibiting *Allivibrio fischeri* and related parameters.

	CTCC	OTCC	M1	M2	M3	M4	M5	M6	M7
C_0_	1.28 × 10^−3^	1.22 × 10^−3^	1.28 × 10^−3^	1.27 × 10^−3^	1.26 × 10^−3^	1.25 × 10^−3^	1.25 × 10^−3^	1.24 × 10^−3^	1.23 × 10^−3^
Molar Ratio (CTCC:OTCC)			12:1	10:3	8:5	1:1	5:8	3:10	1:12
*d*	−0.8941	−0.8026							
*k*	6.634 × 10^−6^	8.775 × 10^−6^							
*R*^2^ (Hill)	0.966	0.954							
RMSE (Hill)	0.031	0.035							
*m*	−0.4371	−80.26	−0.4783	−0.3467	−0.3678	−0.3967	−0.5370	−1.196	−76.32
*a*	1.683 × 10^−6^	−1.791 × 10^−5^	2.085 × 10^−6^	2.240 × 10^−6^	2.658 × 10^−6^	2.633 × 10^−6^	5.823 × 10^−6^	−8.300 × 10^−7^	−4.627 × 10^−5^
*p*	5.156 × 10^−5^	−1.042 × 10^−3^	5.649 × 10^−5^	6.923 × 10^−5^	8.129 × 10^−5^	9.964 × 10^−5^	1.241 × 10^−4^	8.442 × 10^−5^	−1.064 × 10^−3^
*b*	531630	120853	553767	852676	1064074	1326084	196214	182580	43150
*q*	28869	1669	25904	29086	28456	15435	8527	2411	1576
*R*^2^ (BP)	0.999	0.985	0.997	0.992	0.994	0.998	0.996	0.975	0.914
RMSE (BP)	0.022	0.049	0.034	0.058	0.048	0.028	0.033	0.062	0.112
EC_m_	5.75 × 10^−6^	1.24 × 10^−5^	6.00 × 10^−6^	6.00 × 10^−6^	5.50 × 10^−6^	4.75 × 10^−6^	1.55 × 10^−5^	1.15 × 10^−5^	2.70 × 10^−5^
*E*_m_/%	−36.9	−37.1	−40.6	−32.7	−35.8	−35.0	−36.8	−31.0	−39.6
EC_m,SCA_			4.94 × 10^−6^	5.28 × 10^−6^	5.68 × 10^−6^	6.02 × 10^−6^	6.40 × 10^−6^	6.97 × 10^−6^	7.72 × 10^−6^
*E*_m,SCA_/%			−37.5	−37.6	−37.8	−37.9	−38.0	−38.2	−38.5
EC_CP_			3.01 × 10^−6^	1.97 × 10^−5^	1.18 × 10^−5^	1.82 × 10^−5^	1.32 × 10^−5^	3.40 × 10^−5^	2.05 × 10^−5^
*E*_CP_/%			−30.1L	−29.8R	−35.3R	−32.3R	−35.5L	−25.0R	−33.8L
EC_80_	7.93 × 10^−5^	5.20 × 10^−4^	8.83 × 10^−5^	9.73 × 10^−5^	1.10 × 10^−4^	1.52 × 10^−4^	2.24 × 10^−4^	4.99 × 10^−4^	5.80 × 10^−4^
EC_50_	6.11 × 10^−5^	2.81 × 10^−4^	7.13 × 10^−5^	7.72 × 10^−5^	9.02 × 10^−5^	1.16 × 10^−4^	1.61 × 10^−4^	3.04 × 10^−4^	3.24 × 10^−4^
EC_20_	4.81 × 10^−5^	1.58 × 10^−4^	5.35 × 10^−5^	6.29 × 10^−5^	7.52 × 10^−5^	9.07 × 10^−5^	1.19 × 10^−4^	1.84 × 10^−4^	1.93 × 10^−4^
EC_0_	3.89 × 10^−5^	9.90 × 10^−5^	4.41 × 10^−5^	5.31 × 10^−5^	6.55 × 10^−5^	7.27 × 10^−5^	9.16 × 10^−5^	1.16 × 10^−4^	1.30 × 10^−4^
EC_−20R_	2.70 × 10^−5^	5.09 × 10^−5^	3.16 × 10^−5^	3.72 × 10^−5^	5.04 × 10^−5^	4.81 × 10^−5^	5.88 × 10^−5^	5.06 × 10^−5^	7.93 × 10^−5^
EC_−30R_	1.77 × 10^−5^	2.97 × 10^−5^	2.30 × 10^−5^	1.93 × 10^−5^	3.58 × 10^−5^	2.64 × 10^−5^	3.71 × 10^−5^	1.71 × 10^−5^	5.63 × 10^−5^
EC_−30L_	2.88 × 10^−6^	5.28 × 10^−6^	2.95 × 10^−6^	3.52 × 10^−6^	3.42 × 10^−6^	3.38 × 10^−6^	9.44 × 10^−6^	8.28 × 10^−6^	1.24 × 10^−5^
EC_−20L_	1.93 × 10^−6^	2.98 × 10^−6^	2.18 × 10^−6^	2.50 × 10^−6^	2.76 × 10^−6^	2.78 × 10^−6^	7.03 × 10^−6^	4.00 × 10^−6^	7.89 × 10^−6^
EC_−10L_	1.14 × 10^−6^	1.64 × 10^−6^	1.49 × 10^−6^	1.87 × 10^−6^	2.27 × 10^−6^	2.43 × 10^−6^	5.24 × 10^−6^	2.41 × 10^−6^	4.98 × 10^−6^

Note: CTCC is chlortetracycline hydrochloride; OTCC is oxytetracycline hydrochloride; M1–M7 are the mixtures of CTCC and OTCC mixing with the molar ratios of 12:1, 10:3, 8:5, 1:1, 5:8, 3:10, and 1:12 respectively; *R*^2^ is coefficient of determination; RMSE is root-mean-square error; The meanings of parameters *a*, *b*, *p*, *q*, *m*, *k*, and *d* were shown in the Section 3.2. C_0_ is stock concentration; EC_m_ is maximum stimulatory effect concentration; *E*_m_ is maximum stimulatory effect; CP is the cross point between mixture observed J-CRC and CA predicted J-CRC; EC_CP_ is the concentration at CP; *E*_CP_ is the effect at CP; EC_80_, EC_50_, EC_20_, EC_0_ are the 80%, 50%, 20%, 0%-effect concentration respectively; EC_−20R_, EC_−30R_ are −20%, −30%-effect concentration on the right of the lowest point of the J-CRC respectively; EC_−30L_, EC_−20L_, EC_−10L_ are −30%, −20%, −10%-effect concentration on the left of the lowest point of the J-CRC respectively; all the units of C_0_, EC_m_, EC_CP_ and EC*_x_* are mol/L; L and R are on the left and right of the lowest point of J-CRC respectively.

**Table 2:** Joint toxicity effect of chlortetracycline hydrochloride (CTCC) and oxytetracycline hydrochloride (OTCC) to *Allivibrio fischeri*.

Mixtures		EC_80_	EC_50_	EC_20_	EC_0_	EC_−20R_	EC_−30R_	EC_−30L_	EC_−20L_	EC_−10L_
	CTC	96	91	95	93	89	79	101	91	78
	CTC_UL_	100	100	104	103	115	178	131	123	173
	CTC_LL_	81	84	90	85	73	61	61	68	55
M1	Interaction	ADD	ADD	ADD	ADD	ADD	ADD	ADD	ADD	ADD
	CTC	101	97	91	85	81	101	91	84	66
	CTC_UL_	114	105	105	110	NA	NA	155	149	NA
	CTC_LL_	72	85	78	72	62	47	NA	NA	41
M2	Interaction	ADD	ADD	ADD	ADD	ADD	ADD	ADD	ADD	ANT
	CTC	107	97	87	78	65	59	102	81	57
	CTC_UL_	113	106	91	91	108	NA	135	113	NA
	CTC_LL_	84	86	83	71	54	39	NA	50	43
M3	Interaction	ADD	ADD	ANT	ANT	ADD	ANT	ADD	ADD	ANT
	CTC	90	86	81	77	73	84	110	84	55
	CTC_UL_	97	89	88	87	100	NA	128	92	70
	CTC_LL_	75	78	75	71	60	53	NA	74	48
M4	Interaction	ANT	ANT	ANT	ANT	ADD	ADD	ADD	ANT	ANT
	CTC	74	73	71	68	65	64	42	35	27
	CTC_UL_	79	80	78	80	99	NA	55	45	39
	CTC_LL_	60	64	64	59	52	42	NA	26	20
M5	Interaction	ANT	ANT	ANT	ANT	ANT	ANT	ANT	ANT	ANT
	CTC	46	50	56	63	83	150	53	66	62
	CTC_UL_	61	65	79	113	NA	NA	147	144	179
	CTC_LL_	33	38	42	42	41	37	NA	NA	26
M6	Interaction	ANT	ANT	ANT	ADD	ADD	ADD	ADD	ADD	ADD
	CTC	63	68	70	68	60	50	40	36	32
	CTC_UL_	109	111	122	151	NA	NA	141	177	NA
	CTC_LL_	39	36	41	39	30	22	NA	NA	NA
M7	Interaction	ADD	ADD	ADD	ADD	ANT	ANT	ADD	ADD	ANT

Note: M1–M7 are the mixtures of CTCC and OTCC mixing with the molar ratios of 12:1, 10:3, 8:5, 1:1, 5:8, 3:10, and 1:12 respectively; CTC is co-toxicity coefficient; CI is confidence interval; CTC_LL_ is the lower limit of mixture CTC CI; CTC_UL_ is the upper limit of mixture CTC CI; NA is the CI not available; ADD and ANT refer to the additive and antagonistic action respectively.

**Table 3:** Information about antibiotics used in the experiment.

Chemicals	Abbreviation	CAS No.	Molecular Structure	Purity	Molecular Weight
Chlortetracycline hydrochloride	CTCC	64-72-2	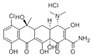	94.6%	515.34
Oxytetracycline hydrochloride	OTCC	2058-46-0	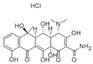	95.6%	496.89

## Abbreviations

CTCC	Chlortetracycline hydrochloride
OTCC	Oxytetracycline hydrochloride
AVF	*Aliivibrio fischeri*
CA	Concentration addition
SCA	Segmented concentration addition
IA	Independent action
CRC	Concentration-response curve
J/U/S-CRC	J/U/S-shaped concentration-response curve
PBZ	Predictive blind zone
CTC	Co-toxicity coefficient
CI	Confidence interval
CTCICI	Co-toxicity coefficient integrated with confidence interval method
EquRay	Direct equipartition ray design
*P_i_*	Component concentration proportion
BP	Biphasic model
BPL	J-CRC left segment model
BPR	J-CRC right segment model
*f*	Function
*R* ^2^	Coefficient of determination
RMSE	Root-mean-square error
EC_m_	Maximum stimulatory effect concentration
*E*_m_	Maximum stimulatory effect
CP	Cross point between mixture observed J-CRC and CA predicted J-CRC
EC_CP_	Concentration at the cross point
*E*_CP_	Effect at the cross point
EC*_x_*	*x*%-effect concentration
EC_−*x*_	−*x*%-effect concentration
EC_−*x*R_	−*x*%-effect concentration on the right of the lowest point of J-CRC
EC_−*x*L_	−*x*%-effect concentration on the left of the lowest point of J-CRC
pEC*_x_*	Negative logarithm of the *x*%-effect concentration
